# Perceptions of Climate Change, Sea Level Rise, and Possible Consequences Relate Mainly to Self-Valuation of Science Knowledge

**DOI:** 10.4236/epe.2016.85024

**Published:** 2016-05-11

**Authors:** Joanna Burger, Michael Gochfeld, Taryn Pittfield, Christian Jeitner

**Affiliations:** 1 Division of Life Sciences, Rutgers University, Piscataway, NJ, USA; 2 Environmental and Occupational Health Sciences Institute, Rutgers University, Piscataway, NJ, USA; 3 Consortium for Risk Evaluation with Stakeholder Participation, Rutgers University, Piscataway, NJ, USA; 4 Environmental and Occupational Medicine, Rutgers Robert Wood Johnson Medical School, Rutgers University, Piscataway, NJ, USA; 5 Ecology and Evolution Graduate Program, Rutgers University, New Brunswick, NJ, USA

**Keywords:** Perception, Decline, Climate Change, Sea Level Rise, Knowledge Level

## Abstract

This study examines perceptions of climate change and sea level rise in New Jersey residents in 2012 and 2014. Different surveys have shown declines in interest and concern about climate change and sea level rise. Climate change and increasing temperatures have an anthropogenic cause, which relates to energy use, making it important to examine whether people believe that it is occurring. In late 2012 New Jersey experienced Super storm Sandy, one of the worst hurricanes in its history, followed by public discussion and media coverage of stronger more frequent storms due to climate change. Using structured interviews, we tested the null hypotheses that there were no differences in perceptions of 1260 interviewees as a function of year of the survey, age, gender, years of education, and self-evaluation of science knowledge (on a scale of 1 to 5). In 2012 460 of 639 (72%) rated “global warming occurring” as “certain” (#4) or “very certain” (#5) compared with 453 of 621 (73%) in 2014. For “due to human activities” the numbers of “certain” or “very certain” were 71% in 2012, and 67% in 2014 and for sea level rise the numbers were 64% and 70%. There were some inconsistent between-year differences with higher ratings in 2012 for 3 outcomes and higher ratings in 2014 for 5 outcomes. However, for 25 questions relative to climate change, sea level rise, and the personal and ecological effects of sea level rise, self-evaluation of science knowledge, independent of years of education, was the factor that entered 23 of the models, accounting for the most variability in ratings. People who believed they had a “high knowledge” (#4) or “very high knowledge” (#5) of science rated all issues as more important than did those people who rated their own scientific knowledge as average or below average.

## 1. Introduction

For at least three decades, agencies, governments, and intergovernmental panels have investigated and documented the changing global climate and sea level rise, determining that these accelerating changes are largely anthropogenic, largely due to the use of fossil fuel [1]. Although there has been controversy, the role of humans, individually and collectively, in affecting climate change is now generally accepted among climate scientists [1]–[3]. The questions for public policy makers, managers, and those connected with energy development and use, therefore, are how rapidly is climate changing, how high will sea level rise, what impacts might occur in a given region (flooding, storms, agriculture), and what can be done to slow the changes or adapt to them. Recently, the New York City Panel on Climate Change [4] noted that sea level rise could be nearly a meter in the next 50 years, which would have severe consequences for New York City, for much of the coastal United States and elsewhere in the world. The large increase in people along coasts worldwide should lead to energy public policies that reduce global warming.

Over half of the US population lives along coasts [5] [6], where severe storms and hurricanes are predicted to increase in number and severity [4] [7] [8]. Damages will be high because of vulnerability to coastal flooding and wind damage [9]. Severe storms threaten property, health, and the lives of people, tax emergency services and infrastructure, and disrupt personal safety and community operations [10]–[12]. Disasters are stressful to individuals and communities [13] [14], and can result in a lack of sufficient clean water, food and heat to survive. Further, severe storms and hurricanes can change the physiognomy of coastal ecosystems, causing harm to individual organisms and habitats [15]–[18]. Both the frequency and intensity of hurricanes and tropical storms, as well as sea level rise, are increasing [19]–[21], which will have a severe impact on ecosystem services [22].

The perceptions and concerns of the public are often an important factor in setting public policy and priorities, and influencing the directions of future commerce, industry, and energy development. Understanding both the perceptions of the public about environmental issues, and why they hold these views is critical to development of sound public policy and to gaining acceptance for such policies. Perceptions of professionals often differ from those of the general public, and may even differ among different professional groups [23]. Perceptions can be elicited in focus groups, or by questionnaires that either ask open-ended questions or ask for a rating (e.g. Likert scale) of the severity or importance of a list of possible concerns. Likert scales have been used to evaluate a variety of environmental concerns, including degradation caused by coastal nature-based tourism [24], environmental quality and eco-cultural attributes [25]–[27], land use [28]–[30], and management and restoration options [31]–[33]. Increasingly, the role of perceptions in climate change issues is being discussed as having an important role in public policy [34] [35].

Several studies have claimed that there is a decreasing interest in climate change [36] [37], and therefore in reducing greenhouse gases, while Krosnick [37] noted that awareness has not declined, but that people were less concerned about climate change than previously. There are thus two different aspects being considered: 1) whether knowledge or awareness of climate change is changing or declining; and 2) whether concern about climate change is changing or declining. The former goes to knowledge, while the latter goes to concern, which eventually translates into public policy. Without concern about climate change, people are less likely to push for public policy to reduce anthropogenic effects on climate [34], and may be more likely to accept adaptation approaches rather than preventive ones.

On 29–30 October 2012 Superstorm Sandy hit the New York/New Jersey coastline. It was the worst storm/hurricane in anyone’s memory. New Jersey and New York bore the brunt of the storm damage, which came ashore on the north Jersey coast, creating record storm surges and rising water, causing 159 deaths and over $70 billion in damages [38]. The present study examines the concerns and perceptions of people living in Central New Jersey about climate change, sea level rise, the potential consequences of sea level rise, and whether these perceptions were different in 2012 and 2014 (before and after Superstorm Sandy). The survey was administered early in 2012 and again in 2014. We test the null hypotheses that there were no differences in perceptions of 1260 people interviewed as a function of year of the survey, age, gender, education, and self-evaluation of science knowledge. Our overall objective was to investigate the relationship between these variables and the ratings or perceptions of climate change and sea level rise and ecological degradation, flooding and other damages. While large, public polls, including those by newspapers, can provide information on overall trends, detailed surveys can provide information not only on whether people believe or are concerned about global climate change, but whether they understand the ecological effects. This study partly addresses the latter issue.

## 2. Methods

The overall protocol was to interview students and others in 2012 and 2014 in central New Jersey, asking them a series of questions about their own knowledge, global climate change and sea level rise, and the possible effects of sea level rise. The study area was centered around Rutgers University in New Brunswick, NJ. All procedures were approved by the Rutgers Human Subjects Review Board. The surveys were voluntary. No individual identifiers were collected on the survey forms, and subjects were informed they could stop the survey at any time. The interview normally required about 10 minutes to complete. The 2012 survey (n = 639) was administered about six months after Hurricane Irene struck New Jersey with heavy rains and flooding and about six months before Superstorm Sandy. The 2014 survey (n = 621) was conducted about 18 months after Sandy.

College students and others were interviewed. Three major aspects were examined: 1) Perceptions about the occurrence of climate change and sea level rise; 2) What factors were causing climate change (e.g. anthropogenic, natural); 3) Perceptions and concerns about how sea level rise will affect economics, recreational opportunities, flooding, and habitat; and 4) Their self-evaluation of science knowledge, and whether they kept current with news. The overall objective was to understand the factors that affect perceptions about climate change and sea level rise, and to see whether views changed from 2012 to 2014, especially given the severity of the Sandy.

The survey had 25 questions, and subjects rated the questions on a Likert scale of 1 (no concern or no knowledge) to 5 (major concern or high knowledge level). Questions related to their knowledge base, science base, whether global warming was occurring or not, whether it was caused by human activities or natural factors, whether sea level rise was occurring in New Jersey, and the world, and whether it affected them and other ecosystem qualities (bird nesting habitat, fish breeding habitat, crab beds, bathable beaches, etc.). Demographic information included age, sex, education (highest grade level), and whether they were science majors.

Differences among groups (years of education, sex, year of interview) were examined using Kruskal-Wallis non-parametric Analysis of Variance. A P < 0.05 was considered statistically significant. We also used General Linear Models [39] to examine the factors that accounted for variation in the Likert rating for the different questions. Age and education were entered as continuous variables. Education was also analyzed as greater or less than 12 years and greater or less than 16 years.

## 3. Results

The two year samples were similar in composition with regard to age, sex, and years of education. The 2012 sample (n = 640) was 55% female, average age 29.8 with 15.4 years of education. The 2014 sample (n = 621) was 49% female, average age 31.5, and 14.0 years of education. The sex, age and education were not significantly different among years.

In 2012, 460 of 639 (72%) rated “global warming occurring” as “certain” (#4) or “very certain” (#5) compared with 453 of 621 (73%) in 2014. For “due to human activities” the numbers of “certain” or “very certain” were 71% in 2012 and 67% in 2014 and for sea level rise the numbers were 64% and 70%. There were few between-year differences, so that we conducted combined as well as separate analyses. Self-rating of scientific knowledge was somewhat higher in 2012 than 2014 (mean 3.26 vs 2.99, P < 0.01). For impacts, there were more between year differences (Table 1) with higher ratings in 2012 for 3 outcomes and higher ratings in 2014 for 5 outcomes.

**Figure 1:**
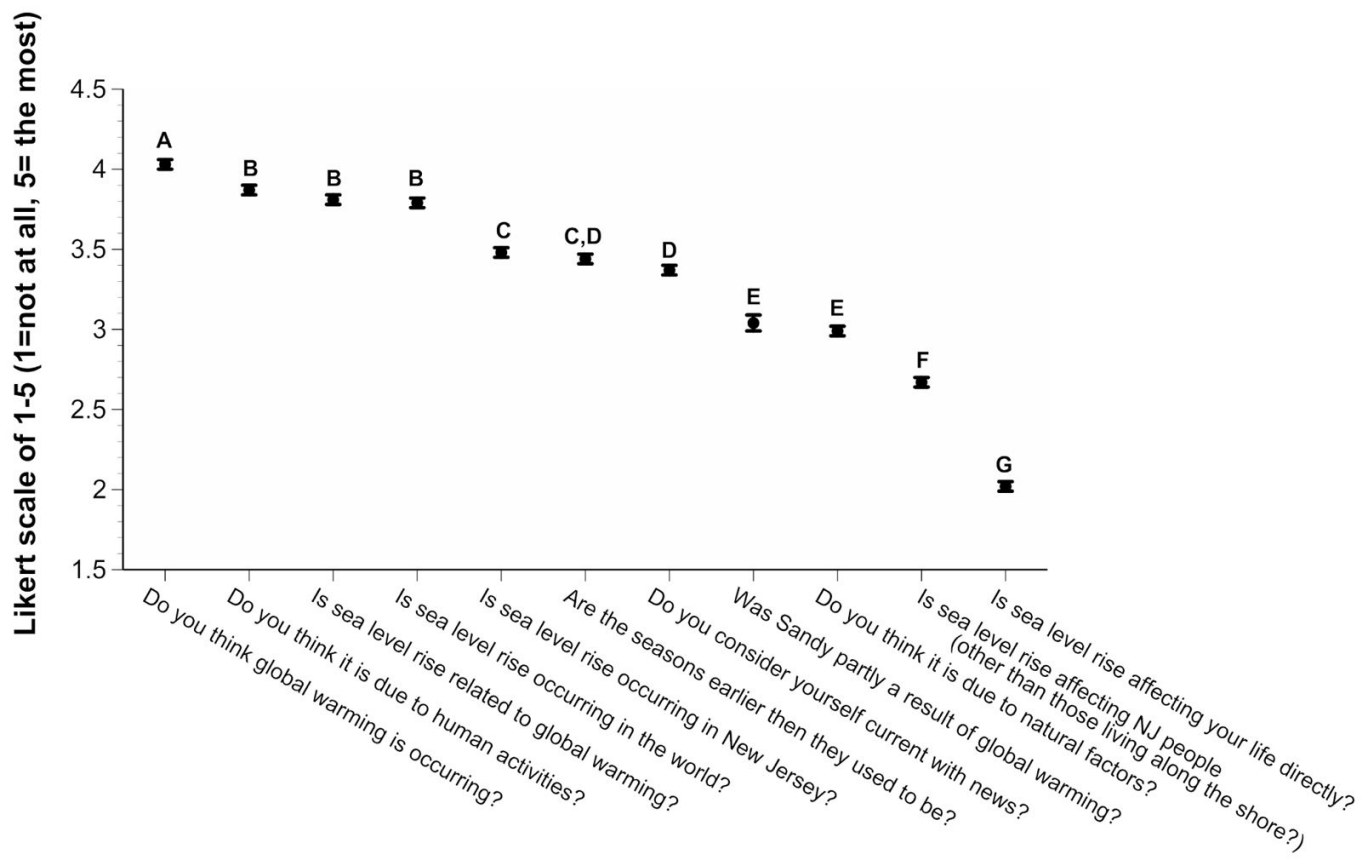
Ratings of New Jersey residents when asked about science knowledge. Letters shown represent groups with no significant difference between them, determined by Duncan’s Multiple Range Test.

**Figure 2:**
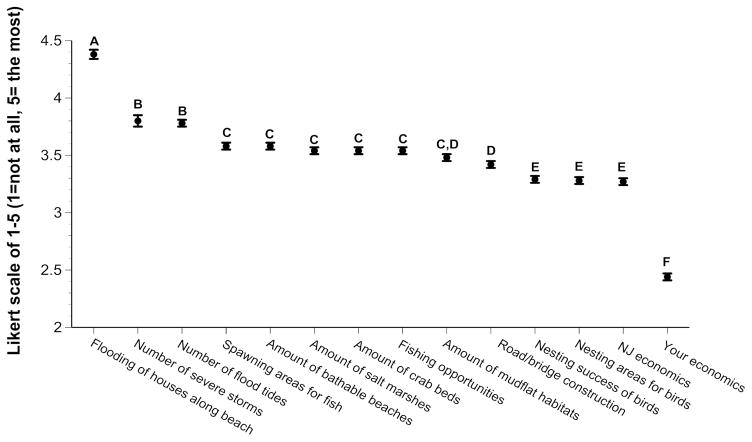
Ratings of New Jersey residents when asked about the effects of sea level rise. Letters shown represent groups with no significant difference between them, determined by Duncan’s Multiple Range Test.

Overall, subjects rated the occurrence of global warming the highest, followed by humans being the cause of global warming, and the occurrence of sea level rise (Figure 1). They rated the effect of sea level rise on their life, and on people living in New Jersey, the lowest, despite the recent occurrence of superstorm Sandy. When asked about the effects of sea level rise, subjects rated flooding of houses along the beach the highest, and their own economics the lowest (Figure 2). Although there were significant differences overall in their rating of other effects, the differences were not great (mean ratings ranged from 3.2 to 3.6).

**Figure 3:**
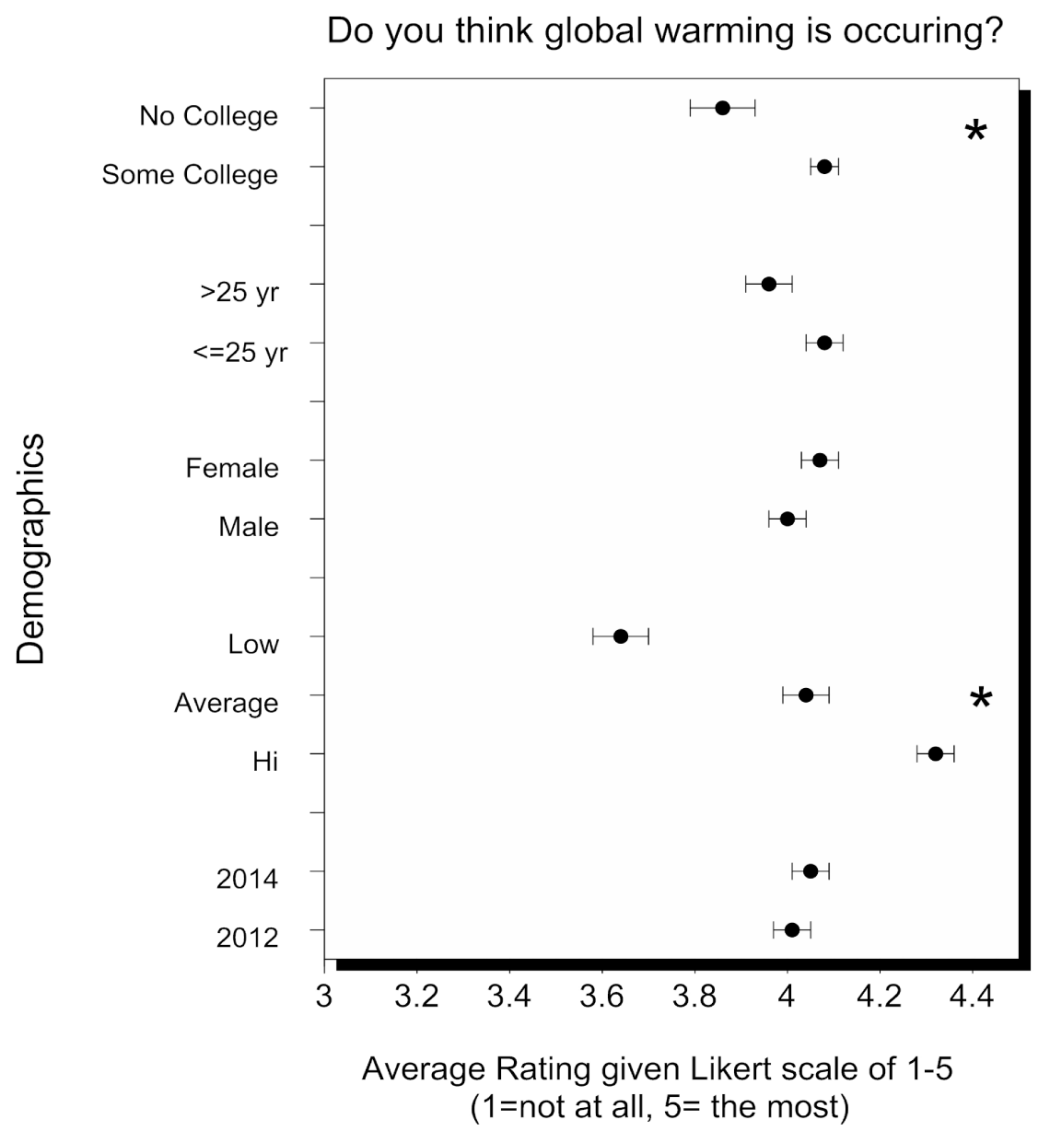
Demographic responses of New Jersey Residents when asked whether they think global warming is occurring. “Low, average, and high” refer to self-assessed knowledge.

Models explaining variation in the responses were significant for all but two of the models, and self-evaluation of science knowledge accounted for the most variability in all of the significant models, followed by year (2012 vs 2014) and education level (Table 1). Figure 3 indicates the mean values for three of the key questions: do you think global warming is occurring? Do you think it is related to human activities? Is sea level rise related to global warming? In all three cases, science knowledge and education were the significant factors, followed by year for sea level rise related to global warming. Values and tests are shown in Table 2 and Table 3. For all questions, subjects who rated their own science knowledge above average rated every question higher (except for whether global warming was due to natural causes, which was not significant). The differences as a function of year (2012 vs 2014) were few, small, and not always in the same direction.

While self-evaluation of science was significantly correlated with all variables, the correlations among variables were not high (R^2^ of 0.06 to 0.15). Thus education itself did not account for the self-evaluation of science level by subjects. The highest associations were between sea level rise and effects variables including directly affecting personal economics (R^2^ 0.40, P < 0.0001).

## 4. Discussion

Since the 1980s, studies in different parts of the world have shown the growing complexity of public and official understanding of climate change, and its causes and impacts, despite a growing scientific certainty about anthropogenic climate change [34] [36]. Krosnick [38] argued that although awareness has not declined, people are less concerned about climate change and greenhouse gases. We are apprehensive that the public discourse has shifted from slowing or preventing anthropogenic climate change (reducing greenhouse gases) to adaptation. In the present study, subjects rated the occurrence of global warming the highest, followed by the occurrence of sea level rise and its relationship to human activities. Thus, we identify four key issues: 1) the public’s awareness of global warming and sea level rise; 2) the public’s knowledge that it is a function of anthropogenic factors; 3) the public’s concern about global climate change; and 4) the public’s understanding or concern about potential effects from global climate change and sea level rise. In addition, temporal aspects also affect public policy. This study, however, examined a population in and around a university community, and was not state-wide or larger. As with all surveys, it reflects the views of the subjects, but there is no reason to assume there were biases in the sampling.

The responses to these questions can be considered representative of an educated population (average 14.7 yrs.) in and around a university community in which 96% of respondents, reported at least 12 years of education. Care should be taken in generalizing more broadly. The US Census Bureau provides data on educational attainment, most recently from a 2009 survey [40]. In the US 85% had a least a high school diploma (12 yrs. of education) and 28% had a bachelors (16 yrs.) or higher. For New Jersey the numbers are 87% and 35%.

### 4.1. Public Awareness of Climate Change and Sea Level Rise

In the present study, “do you think global warming is occurring?” was rated the highest of all questions, indicating that subjects were well aware of global climate change, and thought that it was indeed occurring. Self-evaluation of science knowledge had the greatest effect on ratings about global warming and sea level rise, the relationship between the two, and the relationship between sea level rise and human and ecosystem well-being. People with the highest self-evaluation of science knowledge rated the occurrence of global warming and sea level rise significantly higher than those with average or below average evaluation of their science knowledge. Year, income, age, and sex did not contribute significantly to the variation in rating of the occurrence of global warming or sea level rise.

The strong relationship with self-perceived knowledge is consistent with the growing certainty in the underlying scientific understanding of climate. One could argue that people who think global warming and sea level rise are occurring, as well as those who feel they are not occurring, or are not important or are of no concern, would both feel they had a high degree of scientific knowledge. The correlations between self-knowledge and the other variables were significant, but low (R^2^ of 0.06 to 0.15), indicating that self-knowledge was related to ratings for sea level rise and global warming, but not necessarily to the individual effects for either (e.g. loss of coastal habitats, spawning areas for fish).

Barriers to perceptions of climate change can relate to education, demographics (age, gender) [8], as well as political viewpoints that influence preferences for different sources of information. The natural variation in weather from day to day or month to month [41] may undermine or reinforce views and concerns about climate. That is, if a given day, month or year seems unusually cold, some people comment that global warming must not be happening, despite the growing number of record warmest months in recent years Regional temperature anomalies are partly responsible for this belief, and these anomalies have increased over the last 3 decades [42].

### 4.2. Public Knowledge of the Anthropogenic Factors in Global Climate

Overall rating for human activities causing global warming was high (mean of 3.9 ± 0.03) compared to climate change being related to natural causes (mean of 2.99 ± 0.03). This difference is fairly great. For both responses there was no difference between 2012 and 2014. The significant difference in ratings was a function of selfevaluation of science knowledge. Those with higher self-evaluation rated each higher. This argues for a sound energy development policy by governmental officials.

### 4.3. Public Concern about Climate Change and Sea Level Rise

The present study did not ask subjects directly whether they were concerned about global warming, but rather asked whether sea level rise was affecting their life directly, and whether it affected their economics. The lowest mean ratings of the entire survey were given to these two questions (e.g. mean of 2.0 and 2.4 respectively, see Figure 1 and Figure 2), and the average ratings were way below the other ratings. Ratings for awareness of global warming and sea level rise were high, but their concern for themselves was low. This was surprising given that they rated ecological effects much higher (e.g. means of 3.2 to 3.8). Thus, it appears that the subjects interviewed believe there is global warming and sea level rise, that it is due to human activities, and that it affects ecological habitats, but that it does not affect them directly. The latter may account for a general lack of interest in public policy or pressuring the government to do something about greenhouse gases and global warming.

### 4.4. Public Understanding of How Sea Level Rise (or Climate Change) Affects Them

While the subjects did not rate the impact of global warming and sea level rise high for themselves, they did rate the effects on ecosystems and habitats higher. That is, they rated the effect of sea level rise high on the number of storms and floods, and on habitat. Habitat included spawning areas for fish, amount of beaches and salt marshes, amount of crab beds and mudflats, and nesting areas for birds. Thus, the subjects interviewed did not believe the effects of global warming and sea level rise were personal, except for the fishing opportunities and number of floods.

The findings from the present study are in agreement with Carlton and Jacobson [35], who reported that risks to the physical environment were of greater concern than economic risks. Further, Lieske *et al*. [8] reported that although subjects in focus groups were very concerned about climate change (81%), far fewer (36%) considered themselves to be personally at risk. Partly this represents optimistic bias, the belief by individuals that their situation is always better than that of others in general [43], or that the effects will happen to geographically or temporally distant people and places [2]. This view has been termed “remote indifference” (attributed to poverty reformer Jacob Riis, by Johnson [44]). Lieske *et al*. [8], however, found that these concerns were somewhat related to age, education and gender, but they did not examine self-evaluation of science knowledge (as we did in this study).

### 4.5. Temporal Aspects

There is considerable discussion about whether knowledge and concerns about global warming are increasing or decreasing [8] [34] [45] [46]. Partly the issue revolves around whether there are long-term changes in perception, and whether perceptions change with events or climate anomalies [41]. Public perceptions influence public policy [34], making it important to determine if perceptions are changing. Borick and Rabe [47] reported a rebounding in public opinion in the US about climate change, but longer term trends are still to be shown.

Although year was significant for the ratings of some variables the ratings were higher in 2012 for some and higher in 2014 for others. We had predicted that ratings would be higher in 2014 due to the severity of superstorm Sandy, which devastated New Jersey, causing deaths, billions of dollars in damage, power outages, and disruptions for several months to years [40] [50]. Few New Jerseyians escaped direct impacts of that storm. And it engendered predictions of more storms in the future. However, year entered only about half of the variables examined, and was highly significant only for “are the seasons earlier”. Similarly, education (college vs no college) entered only 12 variables as a significant variable.

### 4.6. Public Policy Implications

Public perceptions are critical for changing public policy [34], and it is important to determine whether changes in perception of climate change are permanent or change with weather anomalies [41]. Changing public policy with respect to a topic such as greenhouse gases and other anthropogenic causes of climate change, is difficult when the causal steps are removed from the general public. The expense required to curb greenhouse gases by governments’ means that such fundamental changes will not occur without public support [34]. The public has a critical role to play, for example in energy conservation, and must be willing to make major behavioral and economic changes. To some extent the doomsday predictions may actually lower public concern or initiative or self-efficacy, while increasing knowledge. The role of self-evaluation of science knowledge is important for educators, managers, and public policy makers because it suggests that providing sufficient information, making information available in different forms of social media as well as educational institutions, and developing informative as well as entertaining social media vehicles is critical to advancing awareness of global warming, sea level rise, and the consequences of both. Appealing to self-evaluation of science knowledge may provide a method of providing scientific information about global warming and sea level rise. In other words, improving how a person views their knowledge about science may have a positive effect on the recognition of global warming, on potential impacts, and on self-interest.

The lack of an age effect for many of the variables suggests that it is not a matter of just reaching out to college-age students, to middle-age people in the work force, or to retirees. Educational materials that capture the interest of viewers should address the entire spectrum of people, which suggests targeted devices for different age groups, both in terms of the message and the means of communication (TV, newspapers, brochures, and social media).

Finding solutions to climate change may be a global, inter-governmental issue [3], but dealing with local flooding and storm damage from sea level rise and climate change is not. Suggesting managed retreat of local communities in response to projected sea level rise requires a sophisticated knowledge of sea level rise and future projections [48]–[51]. Perceptions of sea level rise projections and risk strongly influences willingness to engage in community options, such as managed retreat [52] or construction of storm worthy dune systems or dikes [53].

## 5. Conclusion

Overall, the results of this study show an awareness and belief in global change and sea level rise, and in the potential physical and ecological consequences of them. However, subjects rated the effect of climate change and sea level rise on themselves markedly lower than other effects. This may partly explain a lack of public pressure on officials to deal with greenhouse gases. Thus, self-interest is not yet a motivating factor for their other ratings. We suggest that it is critical to increase both knowledge (which in turn will improve their self-evaluation of science knowledge) of climate change and sea level rise, and increasing awareness of the connections between physical/ecological effects and their own well-being. Accomplishing this will require a multi-dimensional, multi-media approach that targets different age groups. This is not because their views differ, but because they use different media to seek knowledge.

## Figures and Tables

**Table 1:** Climate survey. Demographics of study population in New Jersey (2012, 2104). Given are overall means ± standard error for age, education, and income (N = 1260). NS = not significant. Rating scale: 1 = not at all, 5 = the most. Edu = education level. Sci know = Science knowledge.

Age		30.7 ± 0.43							
Education									
Less than high school		21.1%							
In College or College graduate		66.7%							
Graduate level education		12.2%							
Income (thousands of dollars)		99.8 ± 2.3							
	Model			Parameters in Model					
	P	R-Square	F	Year	Income	Age	Edu	Sex	Sci Know
On a scale of 1 – 5 (1 = not at all, 5 = the most)									
Do you think global warming is occurring?	<0.0001	0.08	13.1				*		***
Do you think it is due to human activities?	<0.0001	0.07	10.6				**		***
Is sea level rise related to global warming?	<0.0001	0.08	12.2	**			***		***
Is sea level rise occurring in the world?	<0.0001	0.09	14			*	**		***
Is sea level rise occurring in New Jersey?	<0.0001	0.044	6.4				*		***
Are the seasons earlier then they used to be?	<0.0001	0.09	14.2	***	*				***
Do you consider yourself current with news?	<0.0001	0.21	37.9			***	***	*	***
Was Sandy partly a result of global warming?	0.003	0.05	3.7						***
Do you think it is due to natural factors?	NS	0.01	1.6		*				
Is sea level rise affecting NJ people other than those living along the shore?	<0.0001	0.05	6.99	*					***
Is sea level rise affecting your life directly?	<0.0001	0.036	5.3	*					***
How will sea level rise affect									
Flooding of houses along beach	<0.0001	0.07	5.7				**	*	*
Number of severe storms	0.0002	0.06	4.9			*			**
Number of flood tides	<0.0001	0.06	8.2						***
Spawning areas for fish	0.0003	0.03	4.3	*					**
Amount of bathable beaches	<0.0001	0.035	5.2	**	*				***
Amount of salt marshes	<0.0001	0.08	12.4		*			*	***
Amount of crab beds	<0.0001	0.05	6.8	*	**				***
Fishing opportunities	NS	0.01	1.97	*					
Amount of mudflat habitats	<0.0001	0.09	13.8	*	*		*	*	***
Road/bridge construction	<0.0001	0.05	8.11	*			*		***
Nesting success of birds	<0.0001	0.04	6.6	*			*		***
Nesting areas for birds	<0.0001	0.06	8.5						***
NJ economics	<0.0001	0.07	11.3	**	*		**		***
Your economics	<0.0001	0.05	6.9	**	***		*		**

**Table 2:** Climate Survey. Given are means ± standard error and Kruskal-Wallis X^2^ (p).

	Overall (n = 1260)	2012 (n = 639)	2014 (n = 621)	X^2^ (p)
On a scale of 1 – 5 (1 = not at all, 5 = the most)				
Do you think global warming is occurring?	4.03 ± 0.03	4.01 ± 0.04	4.05 ± 0.04	NS
Do you think it is due to human activities?	3.87 ± 0.03	3.89 ± 0.04	3.85 ± 0.04	NS
Is sea level rise related to global warming?	3.81 ± 0.03	3.75 ± 0.04	3.87 ± 0.05	6.2 (0.01)
Is sea level rise occurring in the world?	3.79 ± 0.03	3.76 ± 0.04	3.81 ± 0.04	NS
Is sea level rise occurring in New Jersey?	3.48 ± 0.03	3.49 ± 0.05	3.47 ± 0.05	NS
Are the seasons earlier then they used to be?	3.44 ± 0.03	3.7 ± 0.04	3.18 ± 0.05	69.9 (<0.0001)
Do you consider yourself current with news?	3.37 ± 0.03	3.4 ± 0.04	3.33 ± 0.05	NS
How do you rate your science knowledge?	3.13 ± 0.03	3.26 ± 0.04	2.99 ± 0.04	20.0 (<0.0001)
Was Sandy partly a result of global warming?	3.04 ± 0.05		3.04 ± 0.05	
Do you think it is due to natural factors?	2.99 ± 0.03	3 ± 0.04	2.99 ± 0.05	NS
Is sea level rise affecting NJ people other than those living along the shore?	2.67 ± 0.03	2.62 ± 0.05	2.72 ± 0.05	NS
Is sea level rise affecting your life directly?	2.02 ± 0.03	1.97 ± 0.05	2.08 ± 0.05	NS
How will sea level rise affect: (1 = not at all, 5 = the most)				
Flooding of houses along beach	4.38 ± 0.04		4.38 ± 0.04	
Number of severe storms	3.8 ± 0.05		3.8 ± 0.05	
Number of flood tides	3.78 ± 0.03	3.78 ± 0.04	3.79 ± 0.04	NS
Spawning areas for fish	3.58 ± 0.03	3.54 ± 0.04	3.63 ± 0.05	4.1 (0.04)
Amount of bathable beaches	3.58 ± 0.03	3.53 ± 0.04	3.64 ± 0.05	3.8 (0.05)
Amount of salt marshes	3.54 ± 0.03	3.61 ± 0.04	3.46 ± 0.05	6.2 (0.01)
Amount of crab beds	3.54 ± 0.03	3.51 ± 0.04	3.57 ± 0.05	2.8 (0.09)
Fishing opportunities	3.54 ± 0.03	3.48 ± 0.05	3.6 ± 0.05	3.3 (0.07)
Amount of mudflat habitats	3.48 ± 0.03	3.48 ± 0.05	3.47 ± 0.04	NS
Road/bridge construction	3.42 ± 0.03	3.42 ± 0.04	3.43 ± 0.05	NS
Nesting success of birds	3.29 ± 0.03	3.44 ± 0.04	3.14 ± 0.05	22.2 (<0.0001)
Nesting areas for birds	3.28 ± 0.03	3.37 ± 0.05	3.19 ± 0.05	6.8 (0.009)
NJ economics	3.27 ± 0.03	3.25 ± 0.04	3.3 ± 0.05	NS
Your economics	2.44 ± 0.03	2.33 ± 0.05	2.55 ± 0.05	9.6 (0.002)

**Table 3:** Climate Survey. Rating scale: 1 = not at all, 5 = the most. Given are means ± standard error and Kruskal-Wallis X^2^ (p).

	Science Knowledge				

	Overall (n = 1260)	Below Avg. (n = 390)	Average (n = 324)	Above Avg (n = 528)	X^2^ (p)
On a scale of 1 – 5 (1 = not at all, 5 = the most)					
Do you think global warming is occurring?	4.03 ± 0.03	3.64 ± 0.06	4.04 ± 0.05	4.32 ± 0.04	98.5 (<0.0001)
Do you think it is due to human activities?	3.87 ± 0.03	3.55 ± 0.06	3.89 ± 0.05	4.09 ± 0.04	59.2 (<0.0001)
Is sea level rise related to global warming?	3.81 ± 0.03	3.47 ± 0.06	3.78 ± 0.06	4.09 ± 0.04	71.0 (<0.0001)
Is sea level rise occurring in the world?	3.79 ± 0.03	3.4 ± 0.06	3.78 ± 0.05	4.08 ± 0.04	81.6 (<0.0001)
Is sea level rise occurring in New Jersey?	3.48 ± 0.03	3.17 ± 0.06	3.44 ± 0.06	3.73 ± 0.05	49.8 (<0.0001)
Are the seasons earlier then they used to be?	3.44 ± 0.03	3.29 ± 0.06	3.37 ± 0.06	3.6 ± 0.05	17.0 (0.0002)
Do you consider yourself current with news?	3.37 ± 0.03	3.12 ± 0.06	3.36 ± 0.06	3.56 ± 0.05	30.3 (<0.0001)
Was Sandy partly a result of global warming?	3.04 ± 0.05	2.76 ± 0.08	3.18 ± 0.09	3.19 ± 0.08	18.4 (<0.0001)
Do you think it is due to natural factors?	2.99 ± 0.03	2.97 ± 0.06	3.02 ± 0.06	2.99 ± 0.05	NS
Is sea level rise affecting NJ people otherthan those living along the shore?	2.67 ± 0.03	2.36 ± 0.06	2.73 ± 0.06	2.87 ± 0.05	44.5 (<0.0001)
Is sea level rise affecting your life directly?	2.02 ± 0.03	1.8 ± 0.05	2.01 ± 0.06	2.2 ± 0.06	24.7 (<0.0001)
How will sea level rise affect: (1 = not at all, 5 = the most)					
Flooding of houses along beach	4.38 ± 0.04	4.21 ± 0.08	4.41 ± 0.07	4.53 ± 0.05	9.3 (0.009)
Number of severe storms	3.8 ± 0.05	3.54 ± 0.08	3.91 ± 0.08	3.97 ± 0.07	16.2 (0.0003)
Number of flood tides	3.78 ± 0.03	3.49 ± 0.06	3.8 ± 0.05	3.99 ± 0.04	46.5 (<0.0001)
Spawning areas for fish	3.58 ± 0.03	3.41 ± 0.06	3.57 ± 0.06	3.72 ± 0.05	15.0 (0.0005)
Amount of bathable beaches	3.58 ± 0.03	3.4 ± 0.06	3.65 ± 0.06	3.67 ± 0.05	13.6 (0.001)
Amount of salt marshes	3.54 ± 0.03	3.14 ± 0.06	3.57 ± 0.06	3.81 ± 0.04	77.7 (<0.0001)
Amount of crab beds	3.54 ± 0.03	3.29 ± 0.06	3.57 ± 0.06	3.71 ± 0.05	34.5 (<0.0001)
Fishing opportunities	3.54 ± 0.03	3.42 ± 0.06	3.63 ± 0.06	3.57 ± 0.05	6.6 (0.04)
Amount of mudflat habitats	3.48 ± 0.03	3.12 ± 0.06	3.45 ± 0.06	3.76 ± 0.05	73.1 (<0.0001)
Road/bridge construction	3.42 ± 0.03	3.17 ± 0.06	3.47 ± 0.06	3.58 ± 0.05	30.4 (<0.0001)
Nesting success of birds	3.29 ± 0.03	3.04 ± 0.06	3.27 ± 0.06	3.5 ± 0.05	35.1 (<0.0001)
Nesting areas for birds	3.28 ± 0.03	2.88 ± 0.06	3.28 ± 0.06	3.58 ± 0.05	75.1 (<0.0001)
NJ economics	3.27 ± 0.03	2.92 ± 0.06	3.32 ± 0.06	3.5 ± 0.04	64.6 (<0.0001)
Your economics	2.44 ± 0.03	2.16 ± 0.06	2.51 ± 0.07	2.59 ± 0.06	28.4 (<0.0001)
